# Clinical and genetic features of Huntington disease in Sri Lanka

**DOI:** 10.1186/1471-2377-13-191

**Published:** 2013-12-05

**Authors:** Dulika S Sumathipala, Rohan W Jayasekara, Vajira HW Dissanayake

**Affiliations:** 1 Human Genetics Unit, Faculty of Medicine, University of Colombo, Sri Lanka

**Keywords:** Huntington disease, CAG repeats, Autosomal dominant, Penetrant

## Abstract

**Background:**

Huntington disease was one of the first neurological hereditary diseases for which genetic testing was made possible as early as 1993. The study describes the clinical and genetic characteristics of patients with Huntington disease in Sri Lanka.

**Methods:**

Data of 35 consecutive patients tested from 2007 to 2012 at the Human Genetics Unit, Faculty of Medicine, University of Colombo was analyzed retrospectively. Clinical data and genetic diagnostic results were reviewed. Statistical analysis was performed using descriptive statistics.

**Results:**

Thirty patients had fully penetrant (FP) CAG repeat mutations and 5 had reduced penetrant (RP) CAG repeat mutations. In the FP group mean ages of onset and diagnosis were 37.5 and 40.4 years, while in the RP group it was 63.0 and 64.8 years respectively. The age of diagnosis ranged from 15 to 72 years, with 2 patients with Juvenile onset (<20 years) and 3 with late onset (>60 years) Huntington disease. The symptoms at diagnosis were predominantly motor (32/35 -91%). Three patients had psychiatric and behavioral disorders. The age difference between onset and genetic diagnosis showed significant delay in females compared to males (p < 0.05). Twenty two (62.8%) had a positive family history, with 13/22 (59.1%) showing a paternal inheritance of the disease. In both groups, those with a family history had a significantly lower age of presentation (p < 0.05). The mean CAG repeat length in patients with FP alleles was 44.6 ± 5 and RP alleles was 37.2 ± 1.1. Age of onset and CAG repeat length of the *HTT* gene showed significant inverse correlation (p < 0.0005, R^2^ = 0.727).

**Conclusions:**

The clinical and genetic features seen in patients with Huntington disease in the Sri Lankan study population were similar to that previously reported in literature.

## Background

Huntington disease is a progressive neurodegenerative disease. The causative mechanism is dominant inheritance of a mutant (CAG)n triplet repeat expansions [1]. The disease manifests as motor disturbances such as impaired coordination, gait ataxia, chorea, dystonia, bradykinesia and rigidity, cognitive decline and neuropsychiatric symptoms [2]. The age of onset is inversely related to the CAG repeat length. This relationship accounts for 50 – 70% of the age variance but does not provide information on the initial symptoms, course or duration of the disease [3].

Worldwide prevalence of HD is 2.71 per 100,000 with wide geographical variation. Population studies suggest that the mutations originated in Europe. According to a meta analysis performed by Pringsheim *et al* the highest prevalence is seen in populations of Western European descent with a prevalence of 5.7 per 100, 000. Asian prevalence is low with studies indicating a prevalence of 0.4 per 100,000 [4].

This study is the first report on clinical and genetic data characteristics of Huntington patients in Sri Lanka.

## Methods

Records of a total of 35 consecutive patients seen from June 2007 to February 2012 were retrospectively assessed. They were outpatients in our genetics clinic referred by either neurologists or psychiatrists. The patients were undergoing routine genetic diagnostic testing for their disorder. They all gave written informed consent for genetic testing following pre test genetic counseling and were offered post test counseling after test results were known. Ethical approval for the study was obtained from the Ethics Review Committee, Faculty of Medicine, University of Colombo.

The patient sample included symptomatic patients with motor symptoms, cognitive decline, and/or psychiatric symptoms with or without a family history. Clinical data on gender, age of onset of symptoms and signs, age at genetic diagnosis and CAG repeat length were collected.

**Figure 1:**
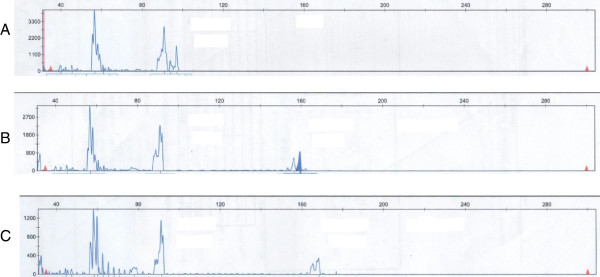
**Electrophoretogram of a patient at risk for HD.** Several shadow bands can be seen around the main allele bands, but based on the intensity measurement the main bands can easily be determined. The internal size control (not shown) allows the accurate sizing of CAG repeat numbers. In this figure **A**, **B** and **C** depict a non-affected individual, a reduced penetrant CAG repeat mutation carrier and a fully penetrant CAG repeat mutation carrier. Bands with 90, 159 and 168 bp were detected and belong to 15, 38 and 41 CAG repeat numbers respectively.

Genetic diagnosis was done by florescent based PCR for detection of the CAG repeat length from the 5’ region of the *HTT* gene. The size of the alleles was determined using internal size markers with relative allele peak area calculation [5]. Figure 1 shows the results of non affected and affected patients in the study population. Alleles with a CAG repeat length of 36 – 39 was defined as reduced penetrant (RP) and ≥ 40 as fully penetrant (FP). Allele length less than 36 was considered nonpathogenic [6].

Statistical analysis was conducted using descriptive statistics, *x*^*2*^ analysis and Student’s t – test. Statistical significance level was set at p < 0.05.

## Results

### HD patients with fully penetrant (FP) CAG repeat alleles

#### Clinical features

Of a total of 35 patients 30 had fully penetrant (FP) CAG repeat alleles, of which 14 (46.7%) were male. Age of onset of the disease was 37.5 ± 10.2 years (range 15–55). The mean age at genetic diagnosis was 40.3 ± 10.6 years (range 15–57) (Table 1). Time period between onset and diagnosis was significantly delayed in FP females compared to males (3.9 ± 3.6 and 1.6 ± 1.2 years) (p < 0.05). Juvenile onset HD was seen in 2 patients, with paternal inheritance and onset in adolescence (ages 15 and 17 years). Clinical symptoms at genetic diagnosis include dystonia and parkinsonian features, typical of juvenile onset HD. Positive family history was seen in 22/30 (70%) of patients with paternal inheritance in 13/22 (62%). A significantly lower age of diagnosis was seen in the presence of a positive family history (p < 0.05).

**Table 1:** Clinical and genetic features of patients with Huntington’s disease

**Patients**	**FP**	**RP**
**N**	**30**	**5**
Mean CAG repeats	44.6 ± 5	37.2 ± 1.1
Sex	M = 21 (46.7%)	M = 1 (20%)
Mean age at onset	37.5 ± 10.2	63.0 ± 6.8
Mean age at diagnosis	40.3 ± 10.6	64.0 ± 6.8
Positive FH	21/30	1/5
Age of diagnosis	40.4 ± 10.8	64 ± 6.8
Positive FH	38.1 ± 11.5	57
Negative FH	45.2 ± 6.6	65.8 ± 6.4
Duration from onset to diagnosis		
Male	1.6 ± 1.1	0
Female	3.9 ± 3.6	1.25 ± 0.5

FP = Fully penetrant allele group.

RP = Reduced penetrant allele group.

N = Numbers, M = Males.

FH = Family History.

### Genetic features

**Figure 2:**
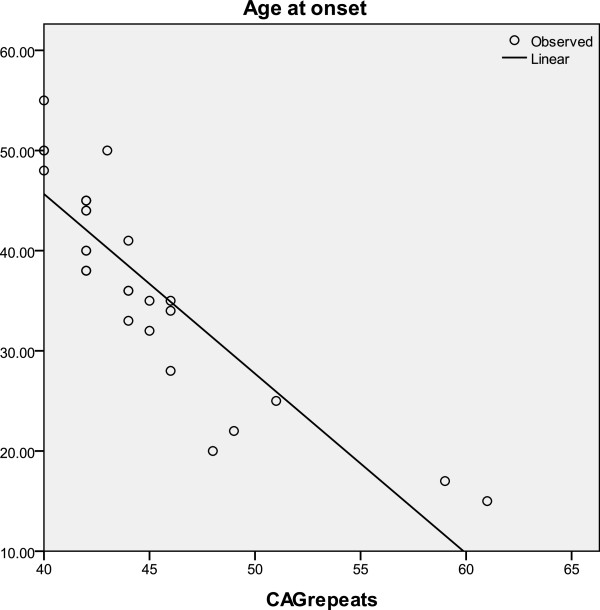
Inverse relationship present between the age of onset of the disease and CAG repeat numbers.

The mean CAG repeat length in the FP patient group was 44.6 ± 5. Heterozygous allele mutation was seen in all positive patients. There was no significant difference in the CAG repeat length in those with and without a positive family history. The two patients with Juvenile HD had CAG repeat lengths of 59 and 61 respectively. The inverse relationship of age at onset and CAG repeat length is shown in Figure 2 with an adjusted R-square value of 0.727.

### HD patients with reduced penetrant (RP) alleles

Reduced penetrant alleles were present in 5 patients, 4 women and one male. Age of onset and age at diagnosis were significantly higher compared to the fully penetrant allele population (p < 0.0005). Positive family history was present in one patient only. Late onset HD (>60 years) was seen in 3 (60%) patients with CAG repeat lengths ranging from 36–38.

### Patients presenting with behavioral symptoms

Three patients presented for genetic testing of HD with a history of behavioral symptoms (3/35 - 8.6%). One patient had reduced penetrant alleles with an age of onset of 56 years while two had FP alleles and ages of onset of 35 and 36 years.

## Discussion

Results in this first study of HD patients in Sri Lanka showed that clinical and genetic data are similar to previous reports. There is, however, a recruitment bias, as this is a retrospective study where only patients referred to the single government based genetic centre situated in Colombo were included. This means that patients from remote regions of the country may not have been referred for diagnosis. Further studies are needed for an epidemiological overview of Huntington disease in Sri Lanka.

### HD with FP alleles

A complex gender based effect on the phenotypic expression of Huntington disease was evidenced by Zielonka *et al*. A more sever phenotype and faster rate of progression was seen in women especially in the motor and functional domains [7]. In our study, however, females had a significantly later presentation to clinic for diagnosis compared to males. This could be either because there was not a rapid progression of disease in these women contrary to previous reports, or because of social factors intervening and delaying presentation in females. Determining the precise cause must be investigated further.

Nine of the thirty patients (30%) did not have a positive family history of HD. This is similar to previous reports, among them Creighton’s and colleagues comprehensive British Columbia population study in which about 25% of affected individuals had a negative family history [8,9]. Possible reasons for negative family history are new mutation, non – paternity including adoption not known to the affected subject, a parent dying prior to the onset of symptoms or failure to diagnose disease in a parent. Our study design did not, however, allow for further investigation of the specific reasons.

### Genetic features

An inverse relationship was present between the age of onset of the disease and CAG repeat numbers. (Adjusted R^2^ squared = 0.727, p < 0.0005), as expected from previous reports, such as 0.85 in a large cohort of Venezuelan kindred [10], and 0.642 in North American, Australian and European populations [4]. Studies analyzing the onset of symptoms in HD have shown a 70% contribution from CAG repeat length. The remaining variations have been shown to be due to modifier genes and environmental factors, which may account for the disparities seen in different populations [10-12]. We did not investigate further genetic and environmental factors which may differentiate the affected from the non – affected subjects in our study population.

### HD with RP alleles

Patients in the RP group of our study population had a lower prevalence of positive family history than the FP group. Ages of onset were also significantly higher. Studies indicate that more than 20% of individuals with reduced penetrant alleles remain undiagnosed due to their mild clinical phenotype [13]. As reported in previous studies, we found a smaller percent with RP in our population (14%) [14]. This indicates that many patients with RP alleles may not be receiving genetic testing in the population.

### Patients presenting with behavioral symptoms

Surprisingly, there were only 8% of patients in our study with predominantly behavioral symptoms. This is much lower than previously reported prevalence, estimated at approximately 23 - 36% [15]. It is thought to have a twofold etiology, biological and psychosocial [16]. Symptoms which include depression, delusions, hallucinations, sexual dysfunction and suicidal ideation are thought to be the earliest presentations of the disease [17]. This low prevalence in our study may indicate the existence of a hidden patient population with behavioral symptoms that are not receiving diagnostic services for Huntington disease. In other words, there may well be a bias caused by lack of identification and diagnosis of the problem. Though studies indicate that behavioral symptoms are the earliest markers of Huntington disease, in our study ages of presentation were not significantly lower than those presenting with motor symptoms [17]. This may be due to the reduced number of patients seen in the study.

## Conclusion

This study shows that correlation of CAG repeat number with age of onset is similar to that seen in populations reported in literature. However, discrepancies with previous reports in age of women presenting for diagnosis and prevalence of behavioral symptoms warrant further studies in the Sri Lanka HD population.

## Competing interest

No financial or non financial competing interest was received for this project.

## Authors’ contributions

DS retrieved the clinical and genetic data, performed the statistical analysis and drafted the initial manuscript. RJ provided review and critique of the manuscript. VD conceived the study and contributed to the design and coordination of the study and reviewed the manuscript. All authors read and approve the final manuscript.

## Pre-publication history

The pre-publication history for this paper can be accessed here:

http://www.biomedcentral.com/1471-2377/13/191/prepub
